# Genotypic and Phenotypic Characterisation of *Staphylococcus aureus* Enterotoxins Using Single-Cell Raman Spectroscopy and Metabolomics

**DOI:** 10.3390/pathogens15030255

**Published:** 2026-02-27

**Authors:** Xiaohui Song, Ziyi Zhang, Taijie Zhan, Li Liu, Xiaoyue Wei, Yang Liu, Jing Tao, Mengjiao Xie, Gege Liu, Duochun Wang, Yu Vincent Fu, Xiaomei Yan, Qiang Wei

**Affiliations:** 1 National Pathogen Resource Center, Chinese Center for Disease Control and Prevention (Chinese Academy of Preventive Medicine), Beijing 102206, China; xhsong77@163.com (X.S.); lyioiyl@163.com (Y.L.); taojing0101@163.com (J.T.); xiemenjao@163.com (M.X.); mel7763_liu@163.com (G.L.); 2 State Key Laboratory of Microbial Diversity and Innovative Utilization, Institute of Microbiology, Chinese Academy of Sciences, Beijing 100101, China; zhangziyi22@mails.ucas.ac.cn; 3 Jiangxi Provincial Key Laboratory of Tissue Engineering, School of Medical and Information Engineering, Gannan Medical University, Ganzhou 341000, China; taijiezhan@163.com; 4 Shandong Provincial Third Hospital, Jinan 250031, China; liuli022400@163.com; 5 National Key Laboratory of Intelligent Tracking and Forecasting for Infectious Diseases, National Institute for Communicable Disease Control and Prevention, Chinese Center for Disease Control and Prevention (Chinese Academy of Preventive Medicine), Beijing 102206, China; weixiaoyue@icdc.cn (X.W.); wangduochun@icdc.cn (D.W.); 6 Department of Preventive Medicine, Medical College of Yanbian University, Yanji 133000, China; 7 College of Life Science, South-Central Minzu University, Wuhan 430074, China; fuyu@mail.scuec.edu.cn

**Keywords:** enterotoxin genotype phenotyping, Raman spectroscopy, metabolome, *S. aureus*

## Abstract

The discrepancy between the genotypic and phenotypic expression of enterotoxins in *S. aureus* had long been a significant challenge in toxin detection. However, the accurate and rapid application of Raman spectroscopy for the genotypic and phenotypic characterisation of *S. aureus* enterotoxins remains problematic. To address this, the present study utilised a single-cell Raman spectra database from 31 *S. aureus* isolates, acquired via a Raman laser tweezer system. When combined with convolutional neural network analysis, this approach achieved an average accuracy of 99.71% for identifying single-gene toxin types and 99.44% for multi-gene toxin types, with an average phenotypic identification accuracy of 98.71%. Notably, the phenotypic identification accuracy for the three strains carrying the *sea* and *seb* genes reached 100%, and the validation accuracy using unknown genotypes and phenotypes exceeded 85%. Furthermore, the CNN analysis identified characteristic spectral peaks for *S. aureus* enterotoxin genotypes at 1663–1665 cm^−1^, 1570 cm^−1^, and 1117–1119 cm^−1^, corresponding to protein α-helices, guanine, and nucleic acid backbones respectively. Representative peaks for the phenotype were found at 1302–1314 cm^−1^ and 912–923 cm^−1^, corresponding to proteins/lipids and polysaccharides, respectively. Representative peaks for different virulence phenotypes carrying multiple enterotoxin genes were located at 1074–1076 cm^−1^, 1253–1255 cm^−1^, 1326 cm^−1^, and 1327 cm^−1^, corresponding to proteins, nucleic acids, and lipids, respectively. Furthermore, metabolomic analysis of three *S. aureus* strains (*sea*+*seb*+, *sea*+*seb*−, *sea*−*seb*+) revealed metabolic differences in fatty acids, purines, phenylalanine, and aspartic acid, consistent with the corresponding distinct Raman spectral peaks (1458, 1179, 1406–1409 cm^−1^). Thus, this study employed *S. aureus* as a proof-of-concept, establishing for the first time a method combining Raman laser tweezers with convolutional neural networks for identifying *S. aureus* enterotoxin genotypes and phenotypes. It clarified the Raman spectral differential peaks and their corresponding biomarkers among five classical enterotoxin genotypes and phenotypic strains, providing a novel approach for accurate toxin typing and virulence characterisation.

## 1. Introduction

Staphylococcus aureus (*S. aureus*) is a ubiquitous Gram-positive pathogen and one of the major causative agents of foodborne illness and healthcare-associated infections [1,2]. The pathogenicity of *S. aureus* is largely determined by the diverse virulence factors it secretes. Among these, staphylococcal enterotoxins (SEs) are key toxins responsible for food poisoning and superantigenic diseases. To date, over 25 enterotoxins and enterotoxin-like proteins have been identified, with five classic enterotoxins classified as SEA, SEB, SEC, SED, and SEE [3]. The expression of enterotoxin genes is regulated by multiple factors, including the agr quorum sensing system, environmental stress, and epigenetic modifications [4]. Consequently, strains harbouring toxin genes may not necessarily express the corresponding enterotoxin. Conversely, cross-reactivity between enterotoxins may yield false-positive phenotypes. This discrepancy between genotype and phenotype complicated the assessment of *S. aureus*’s actual virulence risk, leading to under- or misjudgements in foodborne disease prevention measures.

Therefore, accurate and rapid identification of enterotoxins is crucial for tracing the source of food poisoning and conducting risk assessments. There is an urgent need for a novel technology that combines rapid non-destructive analysis, simultaneous multi-component detection, and the capability for genotype-phenotype correlation analysis, thereby overcoming the technical limitations of existing methods. Traditional methods for identifying *S. aureus* enterotoxin genotypes predominantly employed molecular biology techniques such as gene sequencing and PCR, whilst phenotypic identification often relied on immunological approaches like immunoblotting [5,6]. Although these conventional methods offered targeted specificity, they involve cumbersome, time-consuming procedures and failed to reveal real-time, intracellular virulence expression activities within bacteria. Raman spectroscopy is a molecular structural analysis technique based on the Raman scattering effect. When monochromatic light illuminates a sample, the vast majority of photons undergo elastic scattering (Rayleigh scattering). However, approximately one photon in ten million undergoes inelastic collision with sample molecules, exchanging energy with molecular vibrational or rotational energy levels during scattering. This results in a shift in the scattered light frequency, known as the Raman shift [7]. This shift corresponds to structural information such as the chemical bonds and symmetry of the target molecule, functioning as “a molecular fingerprint” [8]. By detecting and analysing these characteristic shifts, qualitative, quantitative, and structural analysis of substances can be achieved. Raman technology enables the acquisition of chemical fingerprint spectra for the complete molecular composition of individual living bacteria in a label-free, non-destructive manner. In recent years, artificial intelligence had been applied to analyse Raman spectra, enabling microbial identification at the single-cell level [9,10,11]. Single-cell Raman spectroscopy combined with convolutional neural network (CNN) models had achieved precise identification of SE-producing strains, methicillin-resistant *S. aureus* (MRSA) strains, and strains at different growth stages, with accuracies exceeding 93% in each case. This approach offered the potential for rapid analysis of SE phenotypic characteristics [12]. This innovative approach not only facilitated precise diagnosis of *S. aureus* infections, addressing misidentification challenges in clinical settings, but also predicted the enterotoxin genotyping and phenotyping of *S. aureus*. However, research on Raman technology for distinguishing *S. aureus* virulence genotypes and phenotypes remained limited [13,14]. Furthermore, given the non-destructive, universal, and adaptable nature of Raman spectroscopy, this method could be integrated with omics technologies—such as genomic sequencing, transcriptomics, proteomics, and metabolomics analyses—to enhance the reliability of Raman techniques [15].

This study aimed to utilise a laser-trapped Raman spectroscopy system (LTRS) to construct a single-cell Raman spectroscopy database for five classic enterotoxins (*sea*, *seb*, *sec*, *sed*, *see*) of *S. aureus*, encompassing different genotypes and phenotypes. By employing a convolutional neural network (CNN) model to identify distinctive Raman spectral peaks across different genotypes and phenotypes, we elucidated their spectral variations and corresponding metabolic differences. Validation was conducted using unknown enterotoxin-producing strains and metabolomics analysis, ultimately establishing a method for distinguishing *S. aureus* enterotoxin genotypes and phenotypes based on a Raman laser tweezers system and convolutional neural networks.

## 2. Materials and Methods

### 2.1. Experimental Strains

This study encompassed 31 strains of *Staphylococcus aureus* exhibiting distinct enterotoxin genotypes and phenotypic characteristics. These were selected from a collection of 460 strains originating from clinical, food, and environmental sources within the Staphylococcus aureus strain repository of the National Pathogen Resource Centre (NPRC) at the Chinese Centre for Disease Control and Prevention. The strains were identified through a series of processes and methodologies, including PCR, ELISA, and genomic sequencing, ultimately yielding 31 strains displaying varied enterotoxin genotypes and phenotypic traits.

### 2.2. Genotyping

This study conducted whole-genome sequencing on 31 isolated and purified bacterial strains. The entire process was carried out by Meiji Biotechnology Co., Ltd. (Shanghai, China). BLAST alignment was performed for five classic enterotoxin genes: *sea*, *seb*, *sec*, *sed* and *see*.

### 2.3. Enterotoxin Phenotypic Identification

The detection of enterotoxins produced by *S. aureus* was strictly conducted in accordance with the ‘Food Microbiology Testing: *S. aureus* Testing’ (GB4789.10-2016) [16]. Colonies on nutrient agar plates were eluted with 5 mL saline solution and transferred to 60 mL enterotoxin production medium. Incubation was conducted at 37 °C with shaking at 100 rpm for 48 h, followed by centrifugation (8000 rpm, 20 min) and heat inactivation at 100 °C for 10 min. Take 100 μL of diluted supernatant as the sample and proceed according to the instructions for the *S. aureus* Enterotoxin Typing Kit produced by Meizheng Technology Co., Ltd. (Beijing, China). Result interpretation: When the optical density (OD) value of the negative control is less than 0.3 and that of the positive control is greater than 0.5, the threshold is calculated as the average OD value of the two negative control wells plus 0.2. If the sample’s OD value is greater than or equal to T, the sample is positive; if the sample’s OD value is less than T, the sample is negative.

### 2.4. Growth Curve Determination

Research indicates that the expression of SEs is closely associated with the growth phase of *S. aureus*. The temporal expression patterns of various enterotoxin genes at the mRNA level are largely consistent, with expression levels peaking during the late logarithmic phase of bacterial growth before declining rapidly [17]. Consequently, it is necessary to determine the time required for the experimental strains to reach different growth phases. In this study, the experimental strain was cultured overnight, diluted at a ratio of 1:200, and incubated in a growth curve instrument for 48 h. Each experimental group comprised three replicate wells, with the control group using enterotoxin production medium, also comprising three replicate wells. Growth curves were plotted with time on the *x*-axis and OD_600_ values on the *y*-axis.

### 2.5. Raman Spectra Acquisition

All experimental strains were cultured on *S. aureus* enterotoxin production medium (without agar) for 10 h. Following three rinses with ddH_2_O (3000 rpm, 3 min) to remove the medium, the cultures were diluted to 10^5^ CFU/mL in 0.85% NaCl solution. Raman spectra of individual bacteria were captured at 785 nm using a Raman laser tweezers system (LTRS). Spectral calibration was performed using 10-micrometre-diameter polystyrene microspheres for peaks at 620.9, 1001.4, and 1602.3 cm^−1^. Each bacterial spectrum acquisition was set to 25 s to ensure consistency in strain measurements.

### 2.6. Raman Spectroscopy Processing and Modeling

The Raman spectra were converted into ASCII files using Winspecs software. Processing was performed using the proprietary Ramanpro 0.4.2 software package: cosmic ray signals were removed, background values were subtracted from all Raman spectra, spectral smoothing was applied via Savitzky–Golay filtering, baseline correction was achieved through Modpolyfit, and spectral normalisation was subsequently performed using the maximum–minimum normalisation method. Detailed category metrics (including the number of spectra per label), accuracy on the internal test set, precision, recall rate, and F1 score are presented in Supplementary Tables S1 and S2.

For each metabolic label, a total of no fewer than 180 Raman spectra from independent biological replicates were acquired. To rigorously exclude spectrum-level pseudo-replication, the data were partitioned into entirely independent training (approx. 74%) and internal test sets (approx. 26%) at the single-spectrum level. The 1D-CNN model was optimized solely using the training set through a stratified 10-fold cross-validation process, with dropout layers (0.2–0.5) and max-pooling integrated to suppress overfitting. The internal test set was used only for preliminary performance monitoring and not for final evaluation.

The model’s generalization capability was then evaluated using an entirely independent external validation dataset, consisting of clinical isolates representing strain types not included in the training set. This external dataset (=100 spectra per label) was collected from distinct experimental batches and completely isolated in time, space, and biological source from the training pool. Model performance was comprehensively assessed using multiple metrics, including precision, recall, and F1-score, to ensure robust functional discrimination.

The total number of Raman spectra per label for each model is provided in Supplementary Table S1. For each label, the spectra were acquired from multiple independent strains to ensure sufficient representation and diversity for model training and evaluation. This table allows readers to assess the coverage of each label and the number of contributing strains, addressing concerns regarding small sample size and label representation.

It should be noted that for the specific analysis of the three strains harbouring *sea* and *seb* genes (NPRC 1.2.2590: fully expressed; NPRC 1.2.2591: *sea* only; NPRC 1.2.2595: *seb* only), model predictions were performed using only the available spectra from these strains without external validation. Therefore, the reported predictive accuracy for these strains reflects internal classification performance rather than strain-level generalization.

To accurately identify microbial functions and metabolic states, a deep learning framework based on a one-dimensional convolutional neural network (1D-CNN) was developed. The model architecture was optimized for hierarchical feature extraction from the high-dimensional Raman fingerprints 555–1815 cm^−1^. Specifically, the 1D-CNN consists of four convolutional layers with increasing filter counts (32, 64, 128, and 128). The initial layer utilizes a kernel size of 5 to capture broad spectral envelopes, while subsequent layers employ a kernel size of 3 to resolve fine-grained vibrational features. To ensure model robustness and prevent overfitting, max-pooling layers (size = 2) and progressive Dropout layers (0.2, 0.3, 0.3, and 0.4) were integrated after each convolutional block. The flattened features are processed through a dense layer of 256 units (ReLU activation) before the final Softmax output. The training was conducted using the Adam optimizer with a learning rate of 10^−4^ and a categorical cross-entropy loss function. This architecture allows the model to bridge the gap between raw spectral data and complex cellular metabolomics by identifying non-linear patterns in the molecular vibration signatures.

### 2.7. Metabolomics

This study included NPRC 1.2.2590, NPRC 1.2.2591, and NPRC 1.2.2595 for non-targeted metabolomic LC-MS/MS analysis, with 6 biological replicates per group. Raw data underwent peak detection, extraction, alignment, and integration using Progenesis QI, with compound annotation performed via the HMDB database http://www.hmdb.ca/ (accessed on 29 October 2025), Metlin https://metlin.scripps.edu/ (accessed on 29 October 2025), and Meiji’s proprietary library. To eliminate or minimise experimental and analytical errors, pre-processing was applied to the qualified data. This involved removing features with missing values exceeding 20% within each group in the raw data, followed by imputation using the minimum value across all samples. A normalised data matrix was then generated via sum-normalisation. Simultaneously, variables with a relative standard deviation (RSD) exceeding 30% in QC samples were removed. Logarithmic transformation (log10) was applied to the data, yielding the final data matrix for subsequent analysis. Statistical analyses, including PCA and OPLS-DA, were conducted using the ropls package (Version 1.6.2) in R (Version 4.4.3).

## 3. Results

### 3.1. Genotypic and Phenotypic Characteristics of Enterotoxin in Experimental Strains

This study employed the ELISA method to detect the expression of enterotoxin genes in the experimental strains. Results indicated that 19 Staphylococcus aureus strains carried one enterotoxin gene, with 16 expressing it and 3 not expressing it; 12 Staphylococcus aureus strains carried multiple enterotoxin genes, with 4 expressing all genes and 8 expressing some genes. Information on the experimental strains is presented in Table 1.

### 3.2. Growth Curve of Experimental Strains

To determine the growth patterns of *S. aureus* and the peak production of enterotoxins, the strains were continuously cultured for 24 h. The OD_600_ value of the bacterial suspension was measured every 30 min throughout the entire culture process, and growth curves were plotted. Results revealed highly similar growth patterns across all 31 *S. aureus* strains. During the initial 0–3 h of culture, all strains exhibited a lag phase characterised by slow growth. Between 3 and 11 h, OD_600_ values increased exponentially, indicating the logarithmic growth phase with rapid proliferation. From 11 to 24 h, the OD_600_ value stabilised around 1.8. As illustrated in the figure below, the 10 h mark represents the transition point between the logarithmic and stationary phases for *S. aureus*, coinciding with the peak toxin production for enterotoxins.

### 3.3. Detection of S. aureus Producing Different Types of Enterotoxins by Raman Spectroscopy

#### 3.3.1. Identification of Single-Type and Multitype Enterotoxin Genes in *S. aureus* Using Raman Spectroscopy

**Figure 1:**
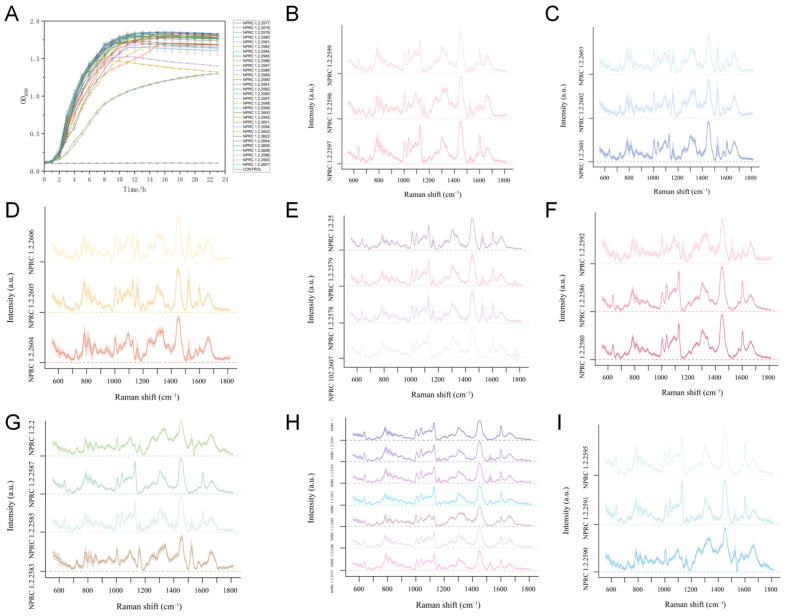
(**A**) Growth curve of the experimental strain; (**B**) Average spectral profile of *S. aureus* carrying *sea* and expressing; (**C**) Average spectral profile of *S. aureus* carrying *seb* and expressing; (**D**) Average spectral profile of *S. aureus* carrying *sec* and expressing; (**E**) Average spectral profile of *S. aureus* carrying *sed* and see genes and expressing; (**F**) Average spectral profile of *S. aureus* carrying enterotoxin genes but not expressing; (**G**) Average spectral profile of *S. aureus* carrying multiple enterotoxin genes and expressing; (**H**) Average spectral profile of *S. aureus* carrying multiple enterotoxin genes partially expressing; (**I**) Average spectral profile of *S. aureus* carrying both *sea* and *seb* genotypes and partially expressing.

**Figure 2:**
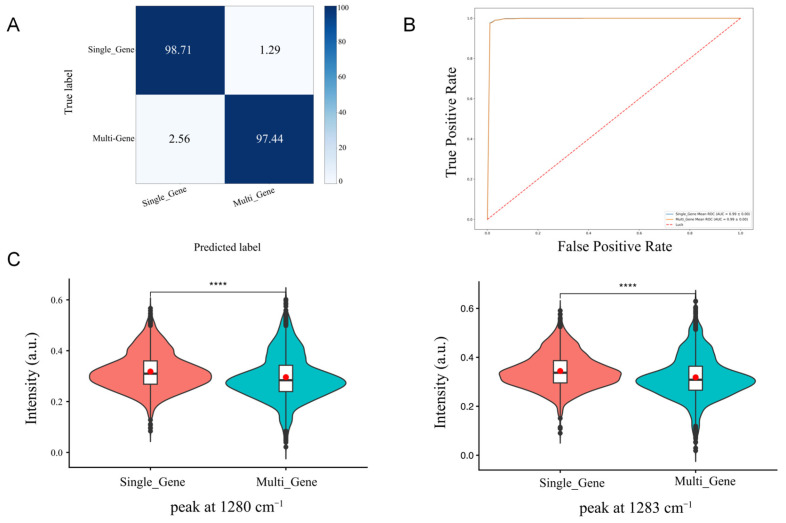
Raman spectroscopy identification of single-type and multi-type enterotoxin genes in *S. aureus*. (**A**) Confusion matrix; (**B**) Receiver operating characteristic (ROC) curve; (**C**) Raman intensity at different peaks and statistical differences. **** *p* < 0.0001. Red dots indicate the mean Raman intensity of each group, while the whiskers denote the minimum and maximum values within 1.5 × IQR.

This study collected Raman spectra from 31 *S. aureus* strains with different enterotoxin genotypes, as shown in Figure 1. To distinguish *S. aureus* strains harbouring single versus multiple enterotoxin gene types, a deep learning model based on single-cell Raman spectroscopy was constructed using a convolutional neural network (CNN) algorithm. The characteristic peaks were shown in Table 2. As depicted in Figure 2A, the single-type identification accuracy reached 98.71%, while multi-type identification achieved 97.44%. This demonstrates that the combination of Raman spectroscopy and CNN constitutes a reliable method for accurately identifying different *S. aureus* subtypes at the species level. To further validate the practicality of this approach, four strains not included in the modelling process (NPRC 1.2.2600, NPRC 1.2.2581, NPRC 1.2.2584 carrying *sea*, *seb*, and *sec*, respectively, and NPRC 1.2.2596 carrying *sea* and *seb*). The model achieved 99% accuracy in identifying *sea* strains, 100% accuracy for *seb* strains, 100% accuracy for *sec* strains, and 66% accuracy for multi-type strains (Figure S1). These results (Figure 2B) demonstrate the high feasibility of this method for detecting single enterotoxin genotypes in *S. aureus*.

Furthermore, this study employed an obscured Raman spectral feature extraction (ORSFE) method to capture specific Raman peaks characteristic of strains harbouring single-type and multi-type enterotoxin genes. Results indicate characteristic peaks at 1279 cm^−1^ (amide III α-helix) and 1280 cm^−1^ (collagen, nucleic acids, and phosphates) for strains harbouring a single enterotoxin gene, while strains carrying multiple enterotoxin genes exhibit characteristic peaks at 938 cm^−1^ (proline, hydroxyproline, and collagen backbone C-C bonds).

#### 3.3.2. Genotypic and Phenotypic Identification of Single-Type Enterotoxin Strains

**Figure 3:**
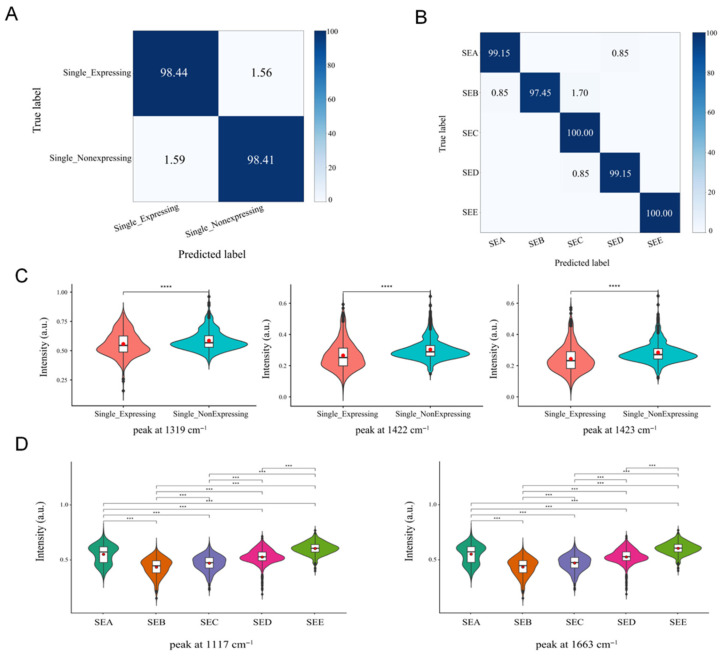
Raman spectroscopy for genotyping and phenotyping of *S. aureus* carrying a single enterotoxin gene. (**A**) Confusion matrix for phenotypic identification; (**B**) Receiver Operating Characteristic (ROC) curve for genotypic identification; (**C**) Raman intensity and statistical significance of phenotypic differential peaks; (**D**) Raman intensity and statistical significance of genotypic differential peaks. ***: *p* < 0.001, ****: *p* < 0.0001. Red dots indicate the mean Raman intensity of each group, while the whiskers denote the minimum and maximum values within 1.5 × IQR.

In this study, a Raman spectroscopy classification model based on a convolutional neural network (CNN) was constructed. The results (Figure 3A) indicate that within a single bacterial strain type, the identification accuracy for the expression group was 98.44%, while that for the non-expression group was 98.41%. Distinctive peaks for the expression group were observed at 1315 cm^−1^ (guanine), 1318 cm^−1^ (protein), 1319 cm^−1^ (guanine), and 1325 cm^−1^; distinctive peaks for the non-expression group were located at 1422–1424 cm^−1^, attributed to deoxyribose (Figure 3C). To validate that the identification results stemmed from genuine spectral differences in the single enterotoxin phenotype, three strains not included in modelling (NPRC 1.2.2600, NPRC 1.2.2581, NPRC 1.2.2584) for validation. The classification accuracies were 97%, 97%, and 94% (Figure S2), respectively, indicating the high feasibility of this method for detecting the enterotoxin phenotype in *S. aureus*.

Subsequently, this study employed the same methodology to analyse strains within the expression group. As shown in Figure 3B, the CNN model achieved a prediction accuracy of 99.15% for strains producing a single enterotoxin type. Specifically, the accuracy for sea was 99.15%, SEB was 97.45%, SEC was 100%, SED was 99.15%, and SEE was 100%. For validation, three strains (NPRC 1.2.2600, NPRC 1.2.2581, NPRC 1.2.2584) were employed, yielding classification results of 100%, 85%, and 100% (Figure S3), respectively. This demonstrates the model’s robust discriminatory capability for distinguishing *S. aureus* strains producing different enterotoxin types. *S. aureus* strains producing different enterotoxin types exhibit distinct characteristic peak positions in Raman spectra. This study employed a masking approach to predict representative Raman peaks for *S. aureus* producing a single enterotoxin type. Results (Figure 3D) indicate that key peaks for sea-producing strains are located at 1068, 1077, and 1079 cm^−1^; for SEB-producing strains at 1045, 1046, 1048, and 1055 cm^−1^; for SEC-producing strains at 1107, 1117, 1118, and 1119 cm^−1^; The key peaks for the SED-producing strain were at 1663, 1664, and 1665 cm^−1^; those for the SEE-producing strain were at 1679, 1688, 1689, and 1690 cm^−1^.

#### 3.3.3. Genotypic and Phenotypic Identification of Multiple Enterotoxin-Producing Strains Based on Raman Spectroscopy

**Figure 4:**
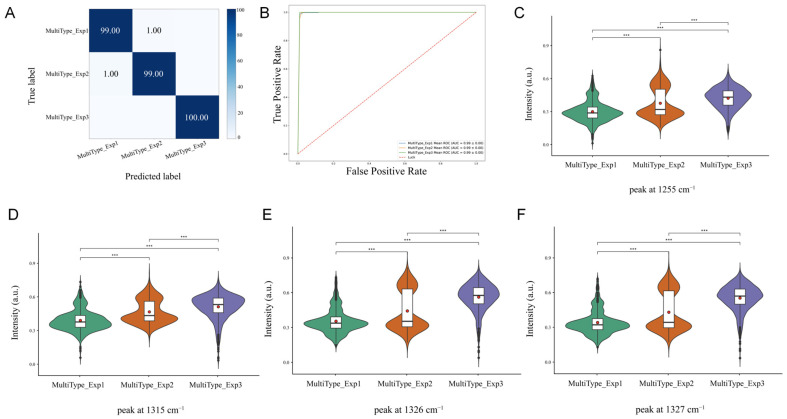
Raman spectroscopy identification of gene expression levels in *S. aureus* carrying multiple enterotoxin genes. (**A**) Confusion matrix; (**B**) Receiver Operating Characteristic (ROC) curve; (**C**–**F**) Raman intensity of differential peaks and statistical significance. *** *p* < 0.001. Red dots indicate the mean Raman intensity of each group, while the whiskers denote the minimum and maximum values within 1.5 × IQR.

This study constructed a Raman spectroscopy classification model based on a convolutional neural network (CNN) to distinguish spectral characteristics of *S. aureus* strains carrying multiple types of enterotoxins. The model demonstrated excellent classification performance on the test set, achieving an overall accuracy of 99.33%. The confusion matrix (Figure 4A) indicates that among strains harbouring multiple enterotoxin genes, the model achieved a 99% prediction accuracy for strains expressing one gene, 99% for strains expressing two genes, and 100% for strains expressing three genes. During validation, the NPRC 1.2.2596 database was employed to assess the model, yielding a classification accuracy of 95% (Figure S4). This demonstrates the CNN model’s capability to efficiently and accurately distinguish Raman spectra of *S. aureus* strains producing different enterotoxin types, exhibiting robust generalisation ability and reliability.

To further elucidate the decision-making mechanism of the model and explore differences between strains expressing varying numbers of genes, this study employed masking techniques to predict representative Raman peaks for strains carrying one, two, or three enterotoxin genes. Analysis results (Figure 4C–F) reveal characteristic peaks at 1068, 1074, 1075, and 1076 cm^−1^ for strains expressing one enterotoxin gene. These positions are typically attributed to the ring breathing vibration of phenylalanine in proteins and the C-O stretching vibration of lipids/carbohydrates. At 1315, 1323, 1326, and 1327 cm^−1^, corresponding to the amide III band vibration associated with the α-helical structure of proteins. At 1182, 1253, 1254, and 1255 cm^−1^, three characteristic peaks associated with enterotoxin genes were identified, corresponding to vibrations associated with proteins and nucleic acids.

#### 3.3.4. Validation of Raman Omics and Metabolomics

**Figure 5:**
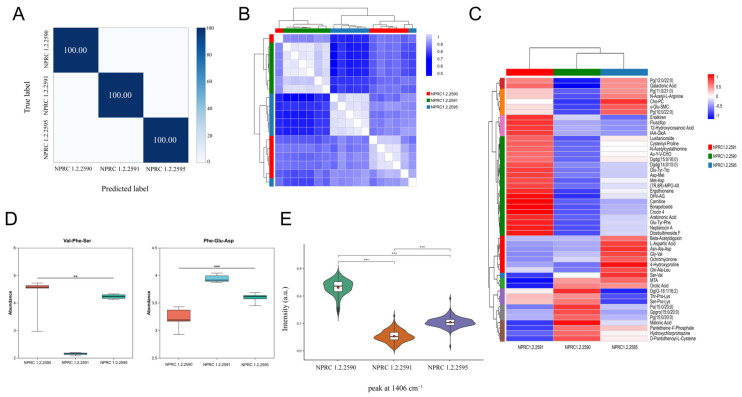
Individuals carrying the *sea* and *seb* genes are identified as *sea*+*seb*+, *sea*+*seb*−, *sea*−*seb*+ Raman spectroscopy and metabolomics of *S. aureus*. (**A**) Confusion matrix; (**B**) Metabolomics sample correlation heatmap; (**C**) Metabolite clustering heatmap; (**D**) Box plot of metabolite distribution for each sample group; (**E**) Raman intensity of differential peaks and statistical significance. ** *p* < 0.01, *** *p* < 0.001.

To further validate the model’s generalisation capability and discriminatory specificity, this study applied it to classify three strains harbouring *sea* and *seb* genes (NPRC 1.2.2590: fully expressed; NPRC 1.2.2591: *sea* only expressed; NPRC 1.2.2595: seb only expressed). As depicted in Figure 5A, the model achieved 100% predictive accuracy for all three strains. To more precisely distinguish phenotypic variations, this study analysed the biological differences between NPRC 1.2.2590, NPRC 1.2.2591, and NPRC 1.2.2595. Characteristic peaks in the strain expressing both *sea* and *seb* genes were concentrated at 1458, 1466, 1467, and 1468 cm^−1^, corresponding to bending vibrations of CH_2_/CH_3_ groups in protein side chains and lipids. Identification of sea-only expressing strains was associated with peaks at 1172, 1178, and 1179 cm^−1^, attributed to ring vibrations of tyrosine and phenylalanine residues in proteins. Discrimination of the seb-expressing strain was highly dependent on characteristic peaks at 1406, 1407, 1408, and 1409 cm^−1^ (Figure 5E), which correspond to symmetric COO^−^ stretching vibrations of aspartic acid and glutamic acid.

To establish a direct correlation between Raman spectral characteristics and metabolic phenotypes, this study employed non-targeted metabolomics to compare the metabolome profiles of the aforementioned three bacterial strains. As depicted in Figure 5B,C, the sample correlation heatmap reveals high intra-sample correlations, whilst the metabolite clustering heatmap clearly demonstrates significant differences in metabolic accumulation patterns among the three groups of strains. These differences align with the classification results from Raman spectroscopy. The results indicate that the fully expressing strain undergoes systemic membrane remodelling in response to dual secretion stress, with characteristic Raman peaks (1458, 1466–1468 cm^−1^) directly corresponding to the significant upregulation of glycerophospholipid metabolism and fatty acid synthesis. The sea-only expressing strain activated de novo nucleotide synthesis pathways to support efficient toxin production, with its discriminative peaks (1172, 1178, 1179 cm^−1^) closely linked to purine/pyrimidine metabolite accumulation. In the seb-expressing strain, core metabolic flux underwent redirection, with characteristic peaks (1406–1409 cm^−1^) originating from specific alterations in aspartate/glutamate metabolism and TCA cycle intermediates (Figure 5D). This finding not only provides direct metabolite-level validation for Raman spectral biomarkers but also reveals the profound potential of Raman spectroscopy as a real-time, non-destructive tool for monitoring pathogen virulence-associated metabolic phenotypes.

## 4. Discussion

Globally, *S. aureus* infections have become a significant public health concern. Consequently, the ability to rapidly and accurately detect the genotypic and phenotypic characteristics of *S. aureus* holds significant public health implications for controlling its transmission and outbreaks [33]. Compared to traditional methods relying on PCR/sequencing for genotyping and ELISA/immunochromatography for phenotyping, the Raman spectroscopy technique developed in this study represents a paradigm shift in detection approaches. Moreover, this approach overcomes the limitations of conventional techniques requiring multiple experiments to characterise genotypic and phenotypic traits, enabling simultaneous identification of both enterotoxin genotypes and phenotypes in *S. aureus* [34,35]. This detection method offers advantages of reduced time consumption, low cost, and non-destructive analysis. It holds significant application prospects in fields such as foodborne disease prevention and control, and clinical infection diagnosis.

In this study, we developed a method combining Raman spectroscopy with machine learning to identify five classic enterotoxin genotypes and phenotypes of *S. aureus*, whilst predicting their biological characteristics at single-cell resolution. This approach achieved outstanding performance in distinguishing single enterotoxin genotypes (98.71%), multiple enterotoxin genotypes (97.44%), and single-type phenotype identification (99.15%). Notably, it demonstrated 100% discrimination accuracy for strains carrying both *sea* and *seb* genes but exhibiting phenotypic differences. single-type phenotype identification (99.15%). Notably, it achieved 100% discrimination accuracy for strains carrying *sea* and *seb* genes but exhibiting phenotypic differences. Representative spectral peaks for different virulence phenotypes carrying multiple enterotoxin genes were observed at 1074–1076 cm^−1^, 1253–1255 cm^−1^, 1326 cm^−1^, and 1327 cm^−1^, corresponding to proteins, nucleic acids, and lipids respectively. During validation, the NPRC 1.2.2596 strain exhibited lower accuracy (66%) in models identifying single and multiple enterotoxin genes, whilst demonstrating high accuracy (95%) in models identifying multiple enterotoxin genes. This discrepancy may arise because strains harbouring multiple enterotoxin genes are regulated by complex mechanisms such as the agr system and environmental signals, necessitating more refined analysis. This further validates the reliability and accuracy of the model for identifying multiple enterotoxin genes. Although the model achieved high overall accuracy, the discrimination performance for multi-toxin genotypes was relatively lower than that for single-toxin genotypes, likely due to overlapping biochemical signatures and increased spectral heterogeneity.

Elucidating the molecular mechanisms underlying the genotypic and phenotypic expression of enterotoxins in *S. aureus* is of paramount importance. In *S. aureus* strains harbouring a single type of enterotoxin gene, peak intensities representing nucleic acids (1280 cm^−1^) and proteins (1279, 1280 cm^−1^) were significantly stronger (*p* < 0.05), potentially reflecting transcription of the enterotoxin gene and synthesis of actively secreted bacterial enterotoxins. Specifically, the 1279 cm^−1^ peak corresponds to amide III (α-helix), while the 1280 cm^−1^ Raman peak is assigned to collagen, nucleic acids, and phosphates, potentially reflecting accumulations of enterotoxin proteins. Strain-specific peaks at 938 cm^−1^ (proline, hydroxyproline, collagen backbone C-C bonds) were statistically significant (*p* < 0.05) in strains harbouring multiple enterotoxin gene types, likely due to increased amino acid production in multi-type enterotoxin-producing strains. These characteristic Raman peak positions provide crucial evidence for identifying enterotoxin genotypes in *S. aureus*. In *S. aureus* strains carrying a single enterotoxin gene type, differential expression peaks were observed at 1315 cm^−1^ (guanine), 1318 cm^−1^ (proteins), 1319 cm^−1^ (guanine), and 1325 cm^−1^. This may occur because during enterotoxin gene expression, the strain’s nucleic acid metabolism or synthesis becomes more active to support the transcription (RNA synthesis) process required for toxin protein synthesis. The differential peak positions in the non-expression group occur at 1422–1424 cm^−1^, attributed to deoxyribose. This may arise because strains not expressing enterotoxin genes are in a quiescent state, where DNA-related metabolism predominates. The molecular content of deoxyribose within the cell remains relatively stable, thus manifesting as a specific differential peak in the Raman spectrum. Specifically, SEA-producing strains exhibit characteristic peaks at 1068, 1077, and 1079 cm^−1^, primarily attributed to proline and nucleic acids. Proline, a key amino acid in bacterial stress metabolism, exhibits concentration changes closely linked to intracellular osmotic pressure regulation triggered by enterotoxin gene expression. The nucleic acid peaks likely arise from enhanced nucleic acid synthesis metabolism during *sea* gene transcription and replication. The SEB-producing strain exhibits characteristic peaks within the 1045–1055 cm^−1^ range, corresponding to proteins and lipids. This may result from *seb* gene expression influencing protein conformational changes and lipid synthesis metabolism; Characteristic peaks for SEC-producing strains cluster at 1107 and 1117–1119 cm^−1^, originating from glucose and C-C stretching vibrations. This likely reflects regulation of the glucose metabolic pathway, altering the content and linkage patterns of cell wall glucose residues, thereby enhancing signals for glucose ring respiratory vibrations and peptidoglycan C-C stretching vibrations; Characteristic peaks of SED-producing strains include 1570 cm^−1^ and 1663–1665 cm^−1^, the former associated with C=N stretching vibrations, the latter belonging to the protein amide I band, indicating that their protein secondary structure may predominantly adopt α-helix or β-sheet conformations. The characteristic peaks of SEE-producing strains are located in the high wavenumber region at 1679 and 1688–1690 cm^−1^. The former is attributed to bound or free NADH, while the latter corresponds to the amide I band. As a core coenzyme in energy metabolism, changes in NADH content reflect the impact of see gene expression on bacterial respiratory chain metabolism. In summary, the differences in Raman characteristic peaks among toxin-producing strains not only reveal alterations in overall cellular molecular composition during enterotoxin production but also provide potential spectral markers for rapid differentiation of *S. aureus* enterotoxin types based on spectroscopy.

In the genotyping of multiple enterotoxin types, characteristic peaks indicative of a single enterotoxin gene expression were identified at 1068, 1074, 1075, and 1076 cm^−1^. When a strain expresses only one enterotoxin gene, cellular transcriptional activity remains at a low activation level. At this stage, conformation changes in nucleic acid phosphodiester bonds are relatively mild, resulting in characteristic spectral peaks within this range. At 1315, 1323, 1326, and 1327 cm^−1^, which correlate with the amide III band vibration of protein α-helix structures. This may arise from significantly heightened translational activity during synthesis of two distinct toxin proteins, where newly synthesised toxins form abundant α-helix secondary structures, amplifying the amide III band signal. At 1182, 1253, 1254, 1255 cm^−1^, characteristic of the expression of three enterotoxin genes. These peaks correlate with both proteins and nucleic acids. This may arise because the transcription of multiple genes demands substantial nucleic acid metabolism support, enhancing base vibration signals. Concurrently, the synthesis of three toxin proteins leads to a substantial accumulation of protein side-chain groups. The Ramanome denotes the aggregate of Raman spectral signals from all biomolecules within a cell, tissue, or biological sample [36]. The metabolome refers to the collection of all low-molecular-weight metabolites present in a cell, tissue, or biological sample under specific physiological conditions, which directly reflect the organism’s physiological and metabolic state. Both focus on intracellular molecular components and metabolic states, jointly elucidating cellular physiological functions and metabolic regulatory patterns. This study employed a strain carrying both *sea* and *seb* genes to conduct simultaneous Ramanomics and metabolomics analyses on different phenotypes. Specifically, the strain expressing only *sea* (NPRC 1.2.2591) exhibited characteristic peaks at 1172 and 1178–1179 cm^−1^ in its Raman spectrum, corresponding to tyrosine, cytosine, and guanine. Metabolomics revealed significantly increased levels of amino acid metabolism-related compounds in this strain, alongside accumulating intermediates involved in energy supply, such as ATP and NADPH. This suggests that the sea-expressing strain may activate specific amino acid metabolism and glycolysis pathways to provide precursors and energy for SEA synthesis. For the *seb*-expressing strain (NPRC 1.2.2595), characteristic Raman peaks were observed at 1406–1409 cm^−1^, a region commonly associated with carboxylate and lipid vibrations. Metabolomic analysis revealed that differential metabolites in this strain were predominantly enriched in lipid metabolism pathways, with significant alterations in free fatty acids and membrane lipid components. This finding confirms at the metabolite level that seb expression is closely linked to bacterial lipid metabolism and remodelling of the cell membrane structure. The strain co-expressing *sea* and *seb* (NPRC 1.2.2590) exhibited significant differences in Raman spectra at 1458 and 1466–1468 cm^−1^, regions representing amide III, adenine, cytosine, and lipids. Metabolomic analysis further revealed that strain NPRC 1.2.2590 exhibited a highly active synthetic metabolic state. Amino acids essential for protein synthesis accumulated significantly, while key cofactors for lipid synthesis—including nucleotide energy molecules and fatty acids—were simultaneously upregulated. This synergistically elevated metabolic pattern provides the metabolic foundation supporting the high energy expenditure and substantial synthetic demands associated with dual toxin co-expression. In summary, Raman spectral characteristics and metabolomics results revealed distinct expression patterns in *S. aureus* harbouring the *sea* and *seb* genes, providing multi-omics evidence for utilising rapid, non-invasive spectroscopic techniques to infer bacterial toxin-producing phenotypes and their underlying metabolic states.

In recent years, although research has attempted to combine spectroscopic techniques with machine learning for microbial identification, most studies have focused on the species or serotype level, with few directly and systematically linking specific virulence factors to their genotypes and phenotypes [37,38,39]. Compared to conventional approaches, this study achieves a shift from population-level averaging to the single-cell level. Raman spectroscopy can capture metabolic state variations among individual cells within a bacterial community under identical culture conditions—a capability unattainable with traditional population-averaged detection methods. Compared to the work of Liu et al. [12], this study not only achieves high-accuracy identification but also, through convolutional neural networks and metabolomics correlation analysis, establishes for the first time a biological link between Raman spectral features and the enterotoxin genotypes and phenotypes of *S. aureus*. Concurrently, biomarkers for *S. aureus* producing different types of enterotoxins have been identified. Furthermore, the Raman database and model constructed for the five classic enterotoxin virulence factors of *S. aureus* form the foundation for the specificity and accuracy of this methodology. This fills a gap in data resources within the field, enabling the model to learn subtle and specific spectral patterns directly related to enterotoxin synthesis regulation, rather than generalised strain differences. However, this study still faces several limitations. Current methods can only achieve qualitative differentiation of toxin types, unable to accurately quantify toxin expression levels, and have limited strain numbers. Future studies should systematically expand the sample size to include diverse Staphylococcus aureus strains with varied enterotoxin genotypes and phenotypes. Additionally, efforts should focus on establishing a quantitative calibration model between toxin concentration and Raman peak intensity, constructing isogenic knockout mutants to confirm the correlation between characteristic peaks and specific toxin genes, and performing blind real-sample testing to validate the model’s practical applicability. Ultimately, the current proof-of-concept study based on 31 bacterial strains should be further developed into a novel, diversity-inclusive rapid identification method for *S. aureus* enterotoxins suitable for real-world applications.

## 5. Conclusions

This study established a Raman spectroscopy-based platform for characterising enterotoxin genotypes and phenotypes using *S. aureus* as proof-of-concept, serving as a detection tool for this bacterium. Integrating Raman spectroscopy with advanced machine learning techniques, the platform rapidly and accurately determines the genotypic and phenotypic characteristics of *S. aureus* enterotoxins at the single-cell level, including the classification of strains carrying single or multiple types of enterotoxin genes. Notably, this approach eliminates the need for multiple independent experiments, as it simultaneously determines multiple genotypic and phenotypic characteristics of *S. aureus* using existing Raman spectral databases and trained models. This methodology provides a rapid and accurate reference for clinical pathogen diagnosis and infectious disease control in related fields. Future work will require expanding the range and quantity of pathogenic bacteria to establish a larger pathogen Raman spectral database, thereby enhancing its applicability [40].

## Figures and Tables

**Table 1:** Basic Information of 31 *S. aureus* Strains.

Grouping	NPRC Number	Source	Year of Isolation	Genotype	Phenotype
Single type	NPRC 1.2.2597	food	2017	*sea*	+
	NPRC 1.2.2598	food	2018		
	NPRC 1.2.2599	food	2018		
	NPRC 1.2.2600	food	2018		
	NPRC 1.2.2601	food	2018	*seb*	
	NPRC 1.2.2602	food	2018		
	NPRC 1.2.2603	food	2019		
	NPRC 1.2.2581	environment	2009		
	NPRC 1.2.2604	food	2019	*sec*	
	NPRC 1.2.2605	food	2020		
	NPRC 1.2.2606	food	2015		
	NPRC 1.2.2584	clinical	2022		
	NPRC 1.2.2578	clinical	2009	*sed*	
	NPRC 1.2.2579	clinical	2009		
	NPRC 1.2.2582	clinical	2009		
	NPRC 1.2.2607	food	2022	*see*	
	NPRC 1.2.2580	clinical	2009	*sea*	−
	NPRC 1.2.2586	clinical	2022	*sec*	
	NPRC 1.2.2592	clinical	2022	*sed*	
Multiple types	NPRC 1.2.2590	clinical	2022	*sea*, *seb*	*sea*+ *seb*+
	NPRC 1.2.2591	clinical	2022		*sea*+
	NPRC 1.2.2596	clinical	2009		*seb*+
	NPRC 1.2.2595	clinical	2024		
	NPRC 1.2.2588	clinical	2022	*seb*, *sec*	
	NPRC 1.2.2589	clinical	2022		
	NPRC 1.2.2577	food	2009	*sec*, *sed*	*sed*+
	NPRC 1.2.2585	clinical	2022	*sea*, *seb*	*sea*+ *seb*+
	NPRC 1.2.2587	clinical	2022		
	NPRC 1.2.2593	clinical	2022	*sea*, *sec*, *see*	*sea*+
	NPRC 1.2.2594	clinical	2022		
	NPRC 1.2.2583	food	2009	*sea*, *seb*, *sed*	*sea*+ *seb*+ *sed*+

+ indicates gene expression; − indicates no gene expression.

**Table 2:** Different Raman peak assignments.

Divide Into Groups	Peak Position (cm^−1^)	Matter	Consult Document
single type	1279	Amide III (a-helix)	[18]
	1280	Amide III & CH_2_ wagging vibrations	[19]
	1283	Differences in collagen content	
Multiple types	938	C-C stretch backboneProline, hydroxyproline, v(C-C) skeletal of collagen backbone	[20]
	941	Skeletal modes (polysaccharides, amylose) Skeletal modes (polysaccharides, amylopectin)	[21]
Single-type expression	1315	Guanine	[22]
	1318	Protein; Amide III	[23]
	1319	Guanine; collagen	[20]
	1325	nucleic acids	[24]
Single type not expressed	1422	Deoxyribose, Adenine (1420 cm^−1^)	[22]
*sea*	1068	Proline (collagen assignment)	[24]
*seb*	1448	Protein/Lipid	[23]
	1455	Lipids (1450 cm^−1^)	[25]
	1117	Glucose; C-C	[23]
	1663	DNA; Proteins, including collagen I	[26]
	1664, 1665	Amide I	[22]
*see*	1679	Bound & free NADH	
	1688	Amide I (1685 cm^−1^)	[27]
	1689	Amide I (1697 cm^−1^)	[28]
Express two enterotoxin genes	1315	Guanine	[23,29]
	1323	Guanine	[23,29]
	1326	CH3CH2 wagging mode in purine bases of nucleic acids (1325–1330)	[30]
	1327	Typical phospholipids; Region associated with DNA & phospholipids; Collagen; Nucleic acids and phosphates (1330 cm^−1^)	[31]
Express three enterotoxin genes	1182	Guanine, adenine (1180–1184 cm^−1^), Cytosine, Thymine (1179 cm^−1^)	[32]
sea, seb expression	1458	Amide III, adenine, cytosine	[19]
	1466	Lipids (1466 cm^−1^)	[26]
	1178	Cytosine, guanine (1179 cm^−1^)	[22]
Express only SEB	1406–1409	COO^−^	[28]

## Data Availability

The original contributions presented in this study are included in the article/Supplementary Materials. Further inquiries can be directed to the corresponding authors.

## Supplementary Materials

The following supporting information can be downloaded at https://www.mdpi.com/article/10.3390/pathogens15030255/s1, Figure S1: Validation of Raman Spectroscopy Identification Model for Staphylococcus aureus Carrying Single and Multiple Enterotoxin Genes; Figure S2: Validation of Phenotypic Identification Model for Staphylococcus aureus Carrying Enterotoxin Gene of Single Type; Figure S3: Validation of Phenotypic Identification Model for Enterotoxin-producing Staphylococcus aureus; Figure S4: Validation of the Gene Expression Quantification Model for Staphylococcus aureus Carrying Multiple Enterotoxin Genes; Table S1: the total number of Raman spectra per label and the number of contributing strains for each model; Table S2: Detailed class-wise performance metrics (precision, recall, F1-score, and support) for each model on the internal test set. Support indicates the number of spectra per label; Table S3: Accuracy and macro-averaged precision, recall, and F1-score were calculated on the internal test set. Test n represents the number of spectra in the internal test set for each model.
