# Supplementary material for: Genotypic and Phenotypic Characterisation of Staphylococcus aureus Enterotoxins Using Single-Cell Raman Spectroscopy and Metabolomics
Source: Pathogens. 2026 Feb 27;15(3):255. doi: 10.3390/pathogens15030255 (PMC13029436; doi:10.3390/pathogens15030255)
Supplement: Supplementary file 1 [file pathogens-15-00255-s001.zip › pathogens-4129600-supplementary.pdf]

Article

Genotypic and Phenotypic Characterisation of *Staphylococcus aureus* Enterotoxins Using Single-Cell Raman Spectroscopy and Metabolomics

Xiaohui Song [1,†], Ziyi Zhang [2,†], Taijie Zhan [3], Li Liu [4], Xiaoyue Wei [5], Yang Liu [1], Jing Tao [1], Mengjiao Xie [1], Gege Liu [1,6], Duochun Wang [5], Yu Vincent Fu [7], Xiaomei Yan [5,*] and Qiang Wei [1,*]

[1] National Pathogen Resource Center, Chinese Center for Disease Control and Prevention (Chinese Academy of Preventive Medicine), Beijing 102206, China; xhsong77@163.com (X.S.); lyioiyl@163.com (Y.L.); taojing0101@163.com (J.T.); xiemenjao@163.com (M.X.); mel7763_liu@163.com (G.L.)

[2] State Key Laboratory of Microbial Diversity and Innovative Utilization, Institute of Microbiology,
Chinese Academy of Sciences, Beijing 100101, China; zhangziyi22@mails.ucas.ac.cn

[3] Jiangxi Provincial Key Laboratory of Tissue Engineering, School of Medical and Information Engineering, Gannan Medical University, Ganzhou 341000, China; taijiezhan@163.com

[4] Shandong Provincial Third Hospital, Jinan 250031, China; liuli022400@163.com

[5] National Key Laboratory of Intelligent Tracking and Forecasting for Infectious Diseases, National Institute for Communicable Disease Control and Prevention, Chinese Center for Disease Control and Prevention (Chinese Academy of Preventive Medicine), Beijing 102206, China; weixiaoyue@icdc.cn (X.W.);
wangduochun@icdc.cn (D.W.)

[6] Department of Preventive Medicine, Medical College of Yanbian University, Yanji 133000, China

[7] College of Life Science, South-Central Minzu University, Wuhan 430074, China; fuyu@mail.scuec.edu.cn

* Correspondence: weiqiang@chinacdc.cn (Q.W.);yanxiaomei@icdc.cn (X.Y.)

† These authors contributed equally to this work.

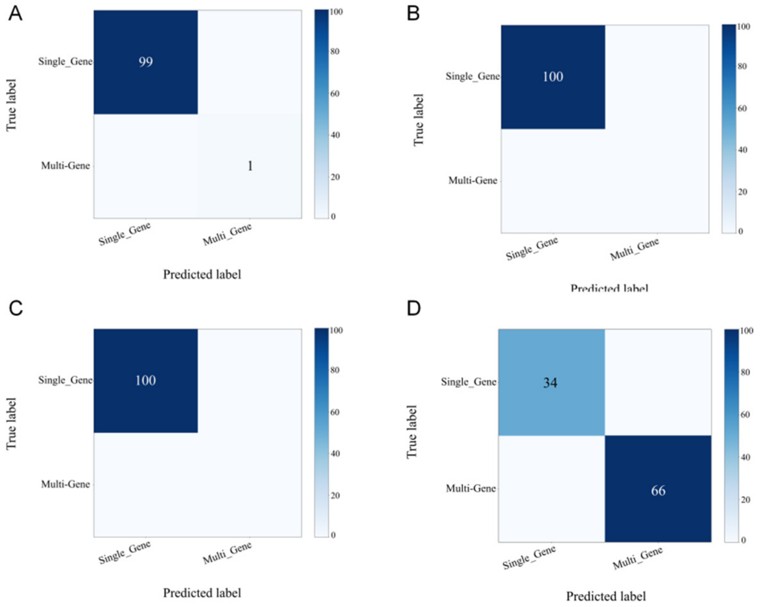

Figure S1 Validation of Raman Spectroscopy Identification Model for Staphylococcus aureus Carrying Single and Multiple Enterotoxin Genes

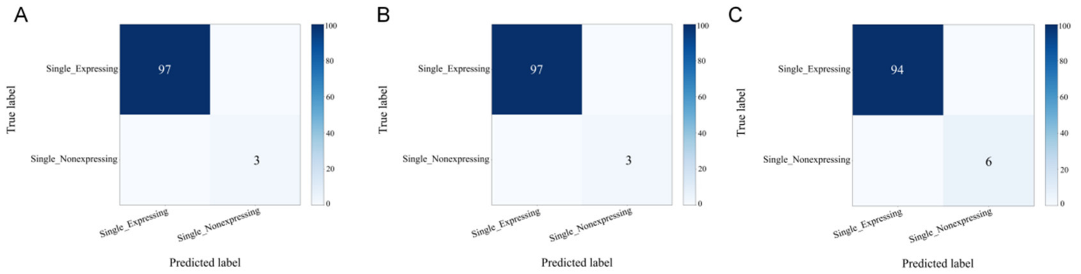

Figure S2 Validation of Phenotypic Identification Model for Staphylococcus aureus Carrying Enterotoxin Gene of Single Type

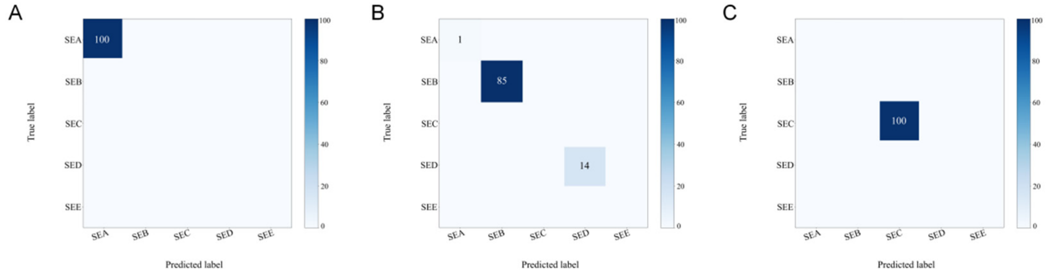

Figure S3 Validation of Phenotypic Identification Model for Enterotoxin-producing Staphylococcus aureus

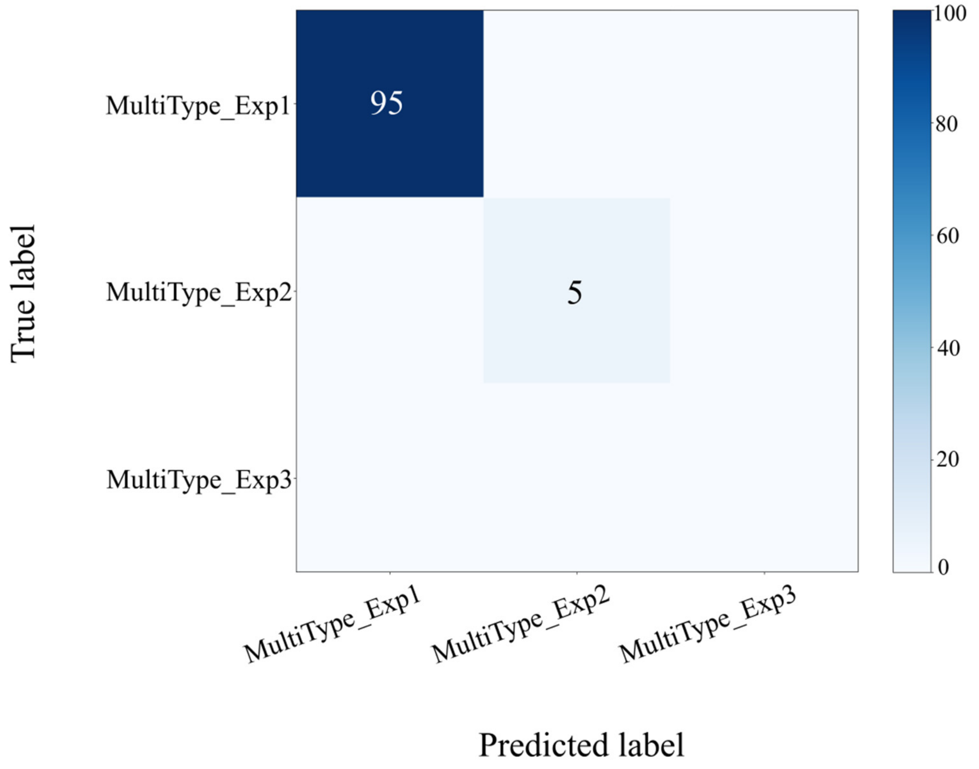

Figure S4 Validation of the Gene Expression Quantification Model for Staphylococcus aureus Carrying Multiple Enterotoxin Genes

Table S1. the total number of Raman spectra per label and the number of contributing strains for each model

Model	Label	Total spectra	Number of strains
Model 1	Multi_Gene	1800	16
	Single_Gene	1800	11
Model 2	Single_Expressing	480	13
	Single_Nonexpressing	480	3
Model 3	SEA	450	3
	SEB	450	3
	SEC	450	3
	SED	450	3
	SEE	450	1
Model 4	MultiType_Exp1	597	7
	MultiType_Exp2	597	3
	MultiType_Exp3	597	1
Model 5	NPRC 1.2.2590	180	1
	NPRC 1.2.2591	180	1
	NPRC 1.2.2595	180	1

Table S2. Detailed class-wise performance metrics (precision, recall, F1-score, and support) for each model on the internal test set. Support indicates the number of spectra per label.

Model	Label	Precision	Recall	F1-score	Support (n spectra)
Model 1	0	0.97	0.99	0.98	464

	1	0.99	0.97	0.98	469
Model 2	0	0.99	0.99	0.99	254
	1	0.99	0.99	0.99	254
	0	0.99	0.99	0.99	117
	1	1.00	0.97	0.99	117
Model 3	2	0.97	1.00	0.99	117
	3	0.99	0.99	0.99	117
	4	1.00	1.00	1.00	117
	0	0.99	0.99	0.99	155
Model 4	1	0.99	0.99	0.99	156
	2	1.00	1.00	1.00	155
	0	1.00	1.00	1.00	47
Model 5	1	1.00	1.00	1.00	47
	2	1.00	1.00	1.00	47

Table S3. Accuracy and macro-averaged precision, recall, and F1-score were calculated on the internal test set. Test n represents the number of spectra in the internal test set for each model.

Model	Classes	Test n	Accuracy	Macro-Precision	Macro-Recall	Macro-F1
Model 1	2	933	0.98	0.98	0.98	0.98
Model 2	2	254	0.99	0.99	0.99	0.99

Model 3	5	585	0.99	0.99	0.99	0.99
Model 4	3	466	0.99	0.99	0.99	0.99
Model 5	3	141	1.00	1.00	1.00	1.00
